# DNA Barcodes for the FIshes of the Narmada, One of India’s Longest Rivers

**DOI:** 10.1371/journal.pone.0101460

**Published:** 2014-07-03

**Authors:** Gulab Dattarao Khedkar, Rahul Jamdade, Suresh Naik, Lior David, David Haymer

**Affiliations:** 1 Paul Hebert Centre for DNA Barcoding and Biodiversity Studies, Dr. Babasaheb Ambedkar Marathwada University, Aurangabad, India; 2 Biodiversity Institute of Ontario, University of Guelph, Guelph, Ontario, Canada; 3 Department of Animal Sciences, R.H. Smith Faculty of Agriculture, Food and Environment, The Hebrew University of Jerusalem, Rehovot, Israel; 4 Department of Cell and Molecular Biology, University of Hawaii, Honolulu, Hawaii, United States of America; Estonian Biocentre, Estonia

## Abstract

This study describes the species diversity of fishes of the Narmada River in India. A total of 820 fish specimens were collected from 17 sampling locations across the whole river basin. Fish were taxonomically classified into one of 90 possible species based on morphological characters, and then DNA barcoding was employed using COI gene sequences as a supplemental identification method. A total of 314 different COI sequences were generated, and specimens were confirmed to belong to 85 species representing 63 genera, 34 families and 10 orders. Findings of this study include the identification of five putative cryptic or sibling species and 43 species not previously known from the Narmada River basin. Five species are endemic to India and three are introduced species that had not been previously reported to occur in the Narmada River. Conversely, 43 species previously reported to occur in the Narmada were not found. Genetic diversity and distance values were generated for all of the species within genera, families and orders using Kimura’s 2 parameter distance model followed by the construction of a Neighbor Joining tree. High resolution clusters generated in NJ trees aided the groupings of species corresponding to their genera and families which are in confirmation to the values generated by Automatic Barcode Gap Discovery bioinformatics platform. This aided to decide a threshold value for the discrimination of species boundary from the Narmada River. This study provides an important validation of the use of DNA barcode sequences for monitoring species diversity and changes within complex ecosystems such as the Narmada River.

## Introduction

Many questions in evolutionary biology, ecology, conservation biology, and biogeography depend on knowledge of species as a biological unit. This makes it essential to critically evaluate methods for determining both the identification of species and species boundaries. Also, in practice, many conservation programs do not adequately address issues relating to intraspecific diversity, in part because of the difficulty in discriminating such variation through morphological analysis [1]. Increasingly, however, both genetic and DNA based tools are making it possible to obtain more detailed and accurate assessments of biodiversity levels both within and between species and to resolve cryptic species complexes. This information will also be essential for identifying conservation units within species [2], [3].

The natural ecology of many river systems makes them an ideal setting for biodiversity studies [4]. Also from an ecological perspective, many of the world’s major rivers are under pressure due to human activities. Asian rivers, in particular those in India, have been heavily impacted in this way. In addition, the impact of climate change and increasing human population density have led to urgent calls for comprehensive biodiversity assessments to provide baseline data on species distributions.

Rivers in India are known to harbor a very diverse fauna. This includes 868 species of freshwater fishes. Of these, 192 are endemic species and 327 species are listed as threatened by the IUCN [5]. This diversity of fishes reflects in part the presence of great riverine systems such as the Narmada, the third longest river in India. Studies on the fish fauna of the Narmada River basin have been conducted by researchers [6]–[8] and by government agencies (CIFRI) during the years 1985 to 1991 [9] using traditional methods of identification based on morphological traits. The first published checklist of fish species by the CICFRI unit from Hoshangabad (1958–66) contained 77 species and the second, conducted by the department of fisheries, Madhya Pradesh, India (1967–71), recorded 46 species. Other studies [6] and [7] recorded totals of 76 species, and a third survey of the Western zone of Narmada fish [8] reported 84 species. Finally, the CICFRI Barrackpore (1991) desk report of the Narmada River listed 95 fish species [10].

However, species identification using these methods can result in misidentification due to high degree of phenotypic plasticity in such characters leading to enlist different species and fluctuations in species numbers. In these cases, alternative tools such as genetic and DNA based markers could help taxonomists to resolve ambiguities to a great extent.

One of these methods, DNA barcoding [11], relies on the sequencing and comparison of a standardized portion of the genome to aid in specimen identification and species discovery. The DNA barcoding method now represents the largest effort to catalogue biodiversity using molecular approaches. Although initially regarded as controversial [12], numerous cases have been reported where the analysis of DNA sequence variation in the cytochrome *c* oxidase subunit 1 (COI) region of mtDNA has proven highly effective for the delineation and identification of animal species in general (see [13] for a review) and fish in particular [14].

Some of the controversies reflect the fact that early barcode studies often examined only a few individuals of each species and were limited in terms of geographic representation [15]–[17]. Although this approach did extend the inclusion of species in databases, it often left gaps in understanding the extent of regional variation in barcode sequences within species, and deciding species boundary [18]. In addition, phylogeographic studies have shown that past geological and climatic events have resulted in population differentiation for freshwater organisms such as fishes because of their limited dispersal ability [19], [20]. Thus, sampling schemes and reference databases must account for these phenomena to permit reliable delineation of species or major lineages. In such situations, new tools such as Automatic Barcode Gap Discovery (ABGD) algorithm have been developed to allow the partitioning of DNA sequence dataset into clusters of like taxa, i.e. candidate or ‘primary’ species by utilizing a range of potential barcode gap thresholds [21]. This approach has been applied to the analysis of specimens from widely dispersed locales [22], [23].

This study aims to first develop a comprehensive DNA barcode library for the fish fauna of the Narmada River. This can improve the quality of future monitoring programs by linking barcode sequences with carefully identified voucher specimens. This study will also provide a better understanding of the genetic variation in fish fauna and the impact of ecological aspects of the river to provide baseline information for creating improved conservation strategies for the Narmada River ecosystem. Furthermore, the information should be more readily available to non- taxonomists, researchers and policy makers to aid in their efforts to establish effective management of this ecosystem.

## Materials and Methods

### Ethical statement

We declare that, the fish under study are not protected under wildlife conservation act and are routinely caught by professional fisherman and sold as a food fish in Indian markets. No specific permit is required for obtaining these fish in India, and no experimentation was conducted on live specimens in the laboratory.

### Sample collection

**Figure 1:**
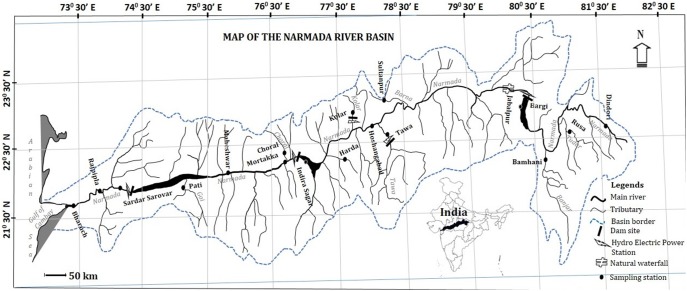
Map Showing sampling sites within the Narmada River basin and its tributaries.

This study examines fish species within the portion of the Narmada River basin that lies between Vindya and Satpura ranges (Figure 1, Table 1). The River has its source near Amarkantak (22°40′0″N to 81°45′0″E) in Madhya Pradesh, and travels 1312 km before it discharges into the Gulf of Cambay in the Arabian Sea (21°39′3.77″N to 72°48′42.8″E). The River is comparatively straight with deep water and hard rocky substrate supporting a rich benthic fauna. Fishes were collected between July 2009 to December 2012 at 17 sites along the main river and its tributaries with ∼100–200 km distance between successive stations (Figure 1; Table S1). Most of the fish specimens were digitally photographed, in case of multiple specimens, representative images were used. Four species that were lacking images include *Acanthophagus latus, Mystus gulio, Hyporamphus dussumieri* and *Parachaeturichthys ocellatus*. (For detailed methodology refer Methods S1)

**Table 1:** Sampling stations on the Narmada River basin.

Sr. no.	Sampling site	Latitude	Longitude	Elevation (m)
1	Dindori	22°56′52.65″	81°04′35.09″	660
2	Rusa	22°32′59.31″	80°44′44.66″	540
3	Bamhani	22°28′49.31″	80°22′59.00″	448
4	Bargi	22°55′10.23″	79°55′9.70″	414
5	Sultanpur	23°06′43.23″	77°57′36.00″	349
6	Tawa	22°33′32.06″	77°57′46.82″	350
7	Hoshangabad	22°45′52.09″	77°42′55.22″	287
8	Kolar	22°57′39.63″	77°20′32.04″	461
9	Harda	22°20′8.59″	77°05′7.07″	284
10	Indirasagar	22°12′46.51″	76°37′46.06″	239
11	Choral	22°14′21.80″	76°03′42.64″	172
12	Mortakka	22°13′29.82″	76°02′59.03″	180
13	Maheshwar	22°10′8.67″	75°35′13.59″	145
14	Pati	21°56′36.54″	74°44′43.66″	199
15	Sardar sarovar	21°52′26.58″	73°41′23.42″	13
16	Rajpipla	21°55′26.80″	73°26‘13.57″	10
17	Bharuch	21°40‘57.74″	72°59‘37.91″	07

### Data analysis

Sequence alignment and assembly was carried out using Codon code Aligner v.3.0.1 (CodonCode Corporation) and MEGA 5 [24]. Sequence divergence values within and among species were employed the Kimura two parameter (K2P) model [25] using analytical functions on BOLD v3.1 (www.boldsystems.org). A neighbor joining (NJ) tree based on K2P distance, nearest neighbor analysis (NN), and nucleotide composition values were also obtained using BOLD. The analysis of genetic distances was complemented by downloading of related sequences from GenBank for comparison with specimens of *Labeo dyocheilus, Puntius sarana, Liza subviridis, Nematalosa japonica and Mystus spp*. These species have deep divergence values that can lead to puzzling identifications. In these and other cases, we have used the ABGD (automated barcode gap discovery) interface web tool available at: http://wwwabi.snv.jussieu.fr/public/abgd/abgdweb.html [21]. For the analysis the ABGD method was first implemented using default parameters and Kimura 2-parameter (K2P) distances to correct for transition rate bias (relative to transversions) in the substitutions [25]. The default for the minimum relative gap width was set to different values between 0 and 1.2. Sequences were aligned and submitted to BOLD project code DBFN and Genbank with accession numbers JX983210–JX983514 (Table S2) (**dx.doi.org/10.5883/DS-NFDB**).

## Results

### Taxon Diversity

**Figure 2:**
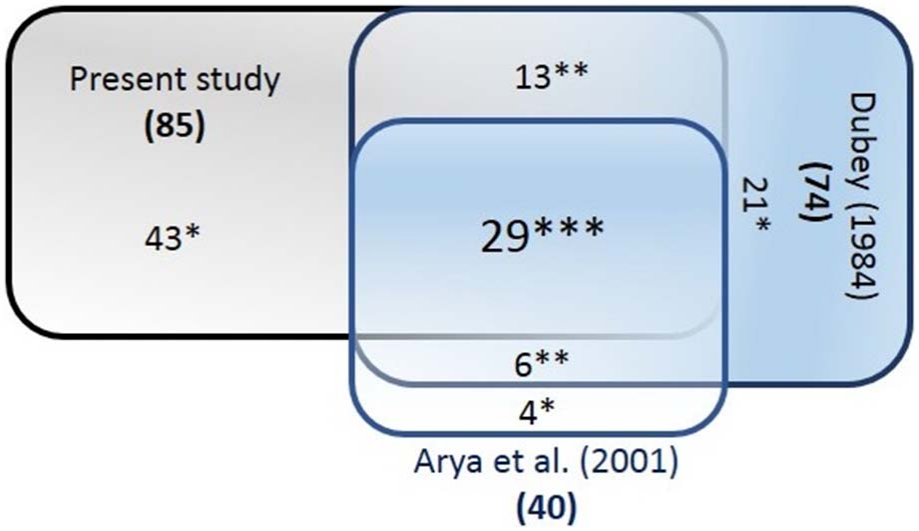
Comparison of fish species with species records from earlier studies. (*specific species to the study; **common species for two studies; ***common species for all studies).

A total of 820 fishes belonging to 90 species, 63 genera, and 34 families (Table S3) were collected at the 17 sites. We generated a total of 314 COI sequences for 83 species (attempts to extract good quality DNA from two species were not successful and did not produce barcodes). The collections included 43 (50%, SE = 0.02) fish species that were not previously known from the Narmada River basin. Three of these taxa could only be identified to a generic level. Also five species endemic to India (*Esomus danricus, Glyptothorax lonah, Mystus montanus, Salmophasia boopis, Scatophagus argus*), and three introduced species (*Cyprinus carpio, Hypophthalmichthys nobilis, Oreochromis mossambicus*) which have not been previously reported from the Narmada River were included in this total. Conversely, 35 (29%, SE = 0.001) species previously reported from the Narmada [26], [6] were not encountered (Figure 2; Table S4).

All amplified sequences were >500 bp (mean, 625 bp) with no insertions, deletions, stop codons and NUMTs. The overall GC content was 45.04% (SE = 0.18) and highest in perches (46.27%; SE = 0.02), followed by cyprinids (44.85%; SE = 0.01) and catfishes (44.27%; SE = 0.02). The mean GC content at codon positions 1–3 was 56.74% (SE = 0.08), 42.9% (SE = 0.03) and 35.17% (SE = 0.29) respectively. Nearly all species exhibited unique barcode haplotypes or cohesive clusters of very closely related haplotypes, which permitted the differentiation of 94% (SE = 0.01) of species. All sequences were submitted to the BOLD project DBFN (**dx.doi.org/10.5883/DS-NFDB**). Four of these represent new records for NCBI Genebank and 12 species for BOLD Systems.

### COI sequence divergence analysis

**Figure 3:**
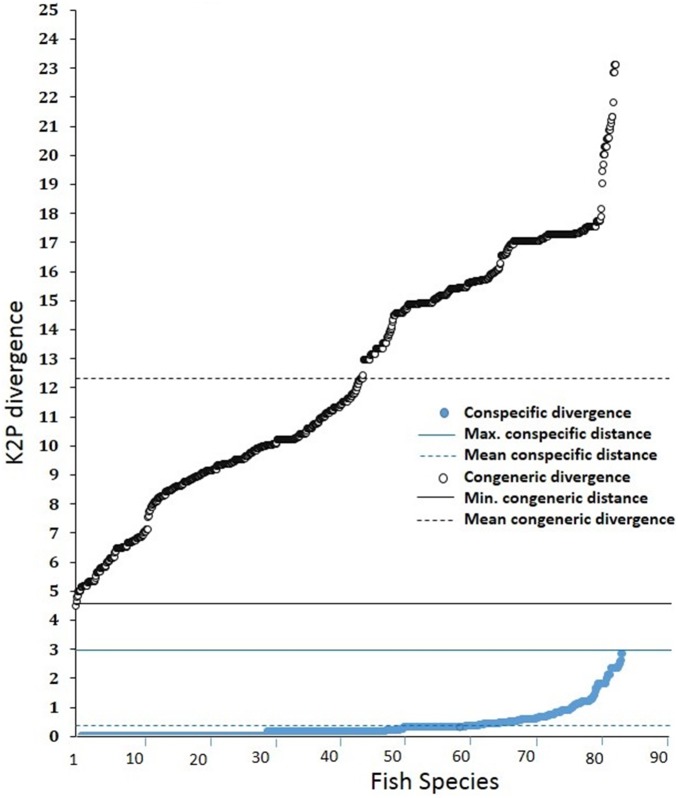
Distribution of conspecific and congeneric K2P mean divergence of 83 fish species from the Narmada River (ascending order). The maximum conspecific divergence (2.9%, blue solid circles) and minimum congeneric divergence (4.66%, black hollow circle) represent the threshold level of conspecific and congeneric divergence respectively. Data series were represented by more than one sequence. 93% of the total 83 species showed divergence below ≤1% and represented true species.

**Figure 4:**
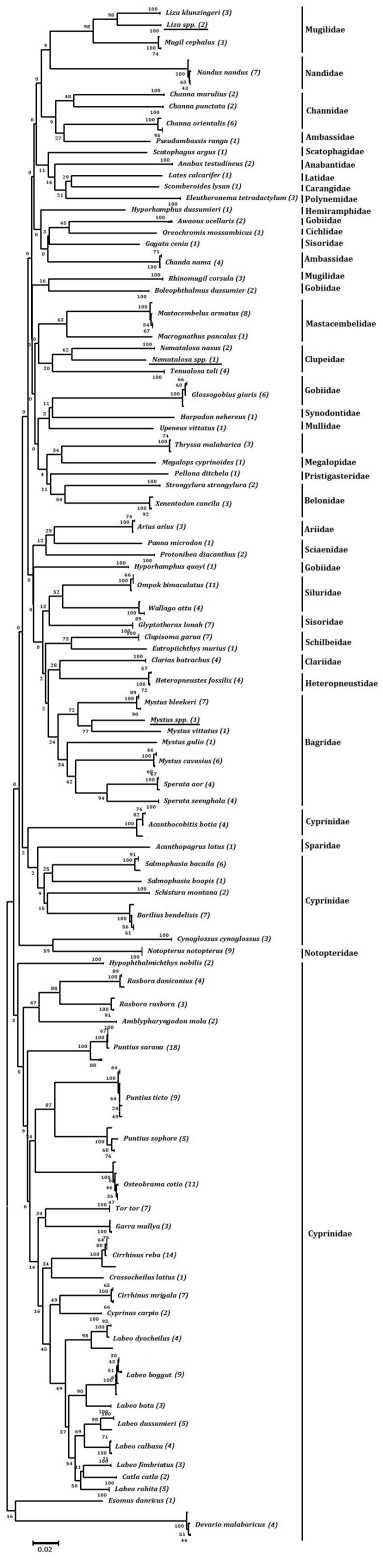
K2P divergence based Neighbor-joining tree of 314 CO1 sequences from 83 fish species from the Narmada River system. (The number of specimens analyzed is shown after each species name).

Out of the 85 species, 83 were well differentiated by COI barcoding with average within species variability of 0.36% (SE = 0.008) compared with 12.29% (SE = 0.06) for species within genera (Table 2 and Figure 3). Values of 17.87% (SE = 0.02) and 22.47% (SE = 0.02) within families and orders, respectively, were also obtained. We were not able to generate barcodes for two species, (*Parachaeturichthys ocellatus* and *Terapon jarbua*). From the values obtained, a steady increase in genetic diversity was observed with increasing taxonomic levels, supporting a marked change in genetic divergence at species boundaries. The average congeneric variability is almost 40 fold higher than the conspecific values, and this also produces a high level of resolution between clusters in the NJ tree to group the species to their corresponding genera and families with sufficient bootstrap support (Table 2, Figure 3 & 4).

**Table 2:** Genetic divergence (K2P) within various taxonomic levels.

				Distance (%)			
	n	Taxa	Comparisons	Min	Mean	Max	SE
Within Species	290	60	856	0	0.36	2.99	0.008
Within Genus	142	10	1003	4.48	12.29	23.12	0.065
Within Family	251	13	9033	4.666	17.87	32.10	0.02
Within Order	123	10	11010	15.836	22.43	32.47	0.025

### Pairwise distances and Automatic Barcoding Gap Discovery (ABGD)

**Figure 5:**
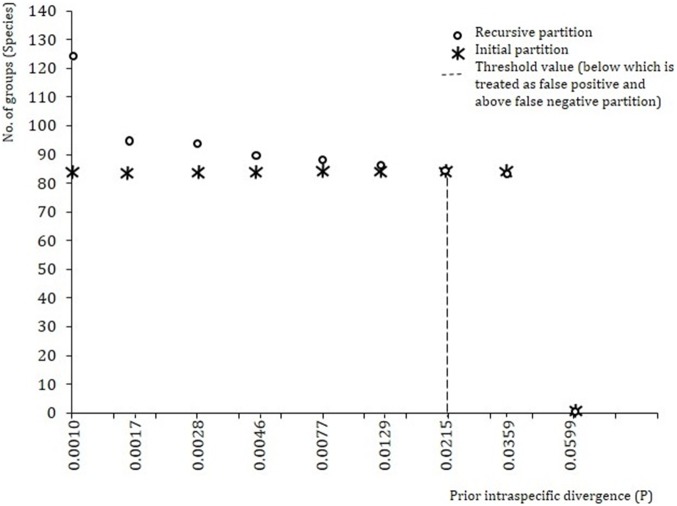
Automatic Barcode Gap Discovery (ABGD) based partition of the data set. We report the number of groups inside the partitions (primary and recursive) as a function of the prior limit between intra- and interspecies divergence. The initial partition is denoted by (o) and recursive portion denoted by (#) and dotted line represents the threshold value (P = 0.0215) for defining species boundary from the Narmada River using COI sequences.

The analysis using the ABGD tool with standard settings at first did not return any results. After lowering the X value (X = relative width of the barcoding gap) to 1.2, the ABGD analysis showed a clustering of the sequences into 8 molecularly defined operational taxonomic units (MOTUs) for the COI (Figure 5). Here, we used a prior intraspecific divergence value of (P = 0.0215, SE = 0.02) which is congruent with the primary species concept. The ABGD results were confirmed independently of the chosen model (Jukes-Cantor and Kimura) and were unaffected by changes of prior limits for intraspecific variation and threshold. The prior maximal distance of P = 0.0215; SE = 0.02 is sufficient to distinguish the fish species in this study (Figure 5). Here, the values below the threshold are treated as false positives since they split real species into two or more partitions. On the other hand greater values (>P = 0.0215, SE = 0.02) are treated as false negatives since these drop the species to a no barcode gap. For example, at a prior maximal distance of P = 0.0215 the *L. dussumier* (NF236) results show congruence with the remaining individuals of this species, but when the prior maximal distance values is lowered (P = 1.29, SE = 0.02), it splits into separate partitions. Considering individual NF236 as a different species to genus Labeo when analyzed, the K2P distance values show a clear overlap between intraspecific (2.19%) and intrageneric (2%) divergences (Table 2). This confirming that, individual (NF236) does not represent a species distinct from *L. dussumier*. This supports the robustness of barcode based delineation of fish species in this study as well as the appropriate use of threshold value.

The nearest-neighbor distance (NND) analysis revealed the closest conspecific individuals to be at an average distance of 0.36% (SE = 0.008) based on a range from 0 to 1% for 93% of the individuals and<3% for the remaining 7% of individuals (Figure 3). The lowest interspecific divergence was observed among *Labeo species* (2.19%; SE = 0.008) and highest in *Channa species* (24%; SE = 0.03).

### Intraspecific divergence and possible hidden taxa

#### i. Labeo dyocheilus

**Figure 6:**
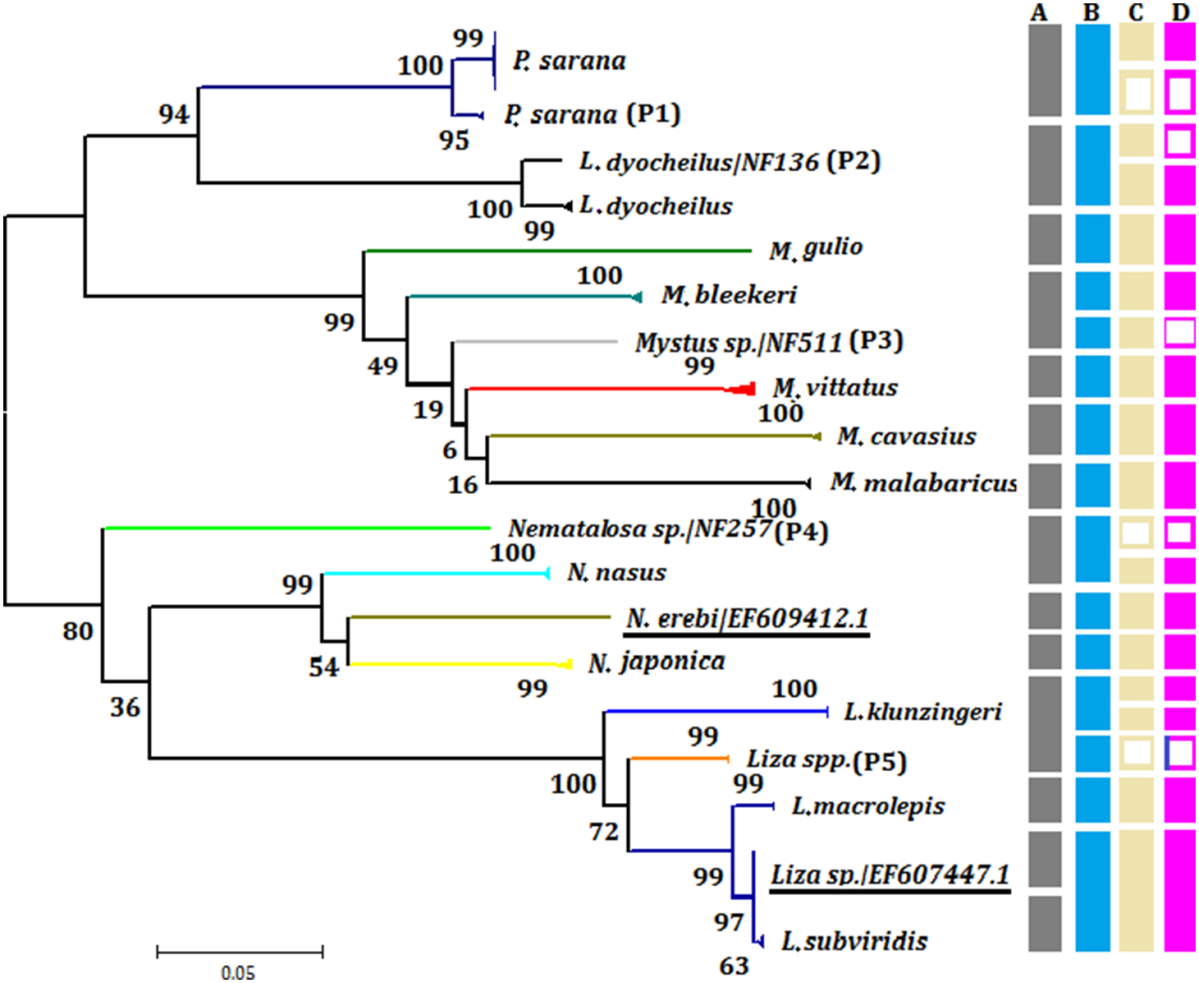
NJ tree based on K2P values showing hidden diversity showing the species having deep conspecific divergence (>2) and various approaches to resolve the putative new species status (Hollow rectangles are treated as confirmation of tested approach). (A) morphological approach (B) Traditional barcode gap approach (>3% divergence or divergence to the magnitude of 10X of mean intraspecific divergence values of nearest species [44] (C) phylogenetics with bootstrap support and (D) ABGD method of recursive partition of sequences into groups using intra and inter specific divergence (P). (Underline represent sequences obtained from NCBI genebank, P1–5 are putative new species).

Genetic divergence (K2P) among individuals of *Labeo dyocheilus* occurring in the Banjar tributary of the Narmada River (Figure 6), was the highest (2.98%) of any region sampled here, indicating the possible presence of sibling species or recently diverged and geographically subdivided populations (voucher ids NF136). Relatively little conspecific variation (0.30% to 0.33%; SE = 0.03) within lineages was observed (Figure 6). When analyzed with AGBD we found optimal threshold level (P = 0.0215, SE = 0.03) to infer NF136 as a putative new species of genus Labeo from the Narmada River. NJ tree analysis showing higher boot strap values are also in confirmation with this new lineage (Figure 6).

#### ii. Puntius sarana

*Puntius sarana* at the Dindori sampling station also exhibited extensive divergence (2.35%; SE = 0.07), forming subclusters in the NJ tree (Figure 6) with intercluster values ranging from 0 to 1.89% (SE = 0.04). This also suggests the presence of possible sibling species or recently diverged geographically subdivided populations (voucher ids NF115 & NF98). To confirm this, we have analyzed all the individuals of this species using ABGD threshold values for partitioning (P = 0.0215; SE 0.02) and bootstrap analysis. This is consistent with the suggestion that NF115 and NF98 are putative sibling species within the genus Puntius (Figure 6).

#### iii. Liza species

Similarly *t*he *Liza sp*. collected from the Bharuch estuary in the Gulf of Cambay shows 6% divergence when compared with the sister species *Liza klunzingeri* (avg. diveregence 0.98%; SE = 0.1) and the sequences obtained from GenBank as *Liza sp.* (avg. divergence 0.43%; SE = 0.001). The rest of species also show extensive divergence (*Liza subviridis,* 0.64%**–**0.68%, SE = 0.03; *Liza macrolepis,* 0.72%**–**0.74%, SE = 0.1). The ABGD analysis further clarifies the species partitioning at an optimum threshold value (P = 0.0215; SE = 0.02). Here, four partitions are formed, and this supports NF550 and NF565 as putative new species (Figure 6). This analysis also clearly indicates the species downloaded for analysis from NCBI Genebank (EF607446.0 and EF607447.1) recorded as Liza spp. were not different from *Liza subvirdis* as the threshold values and bootstrap support can not partition them separately (Figure 6).

#### iv. Nematalosa species

The *Nematalosa* sp. (NF257) collected from Hoshangabad shows 10% genetic variation, whereas sequences from the sister species *Nematalosa nasus* shows an average divergence of 0.173% (SE = 0.09). Comparatively, GenBank sequences of *Nematalosa nasus* (HQ231349.1, HQ231350.1) and *Nematalosa erebi* (EF609412.1) showed an average genetic distance of 0.193% (SE = 0.05) with *Nematalosa japonica* (AP009142.1, EF607513.1) 0.181 (SE = 0.1) (Figure 6). The ABGD based analysis shown for a threshold value (P = 0.01**–**1.00, SE = 0.02) can partition these four species accurately and suggests that NF257 may be a sibling species of the genus Nematolosa.

#### v. Mystus species

The species belonging to the genus Mystus are native to India. Four species of *Mystus* (*M. bleekeri, M. cavasius, M. vitatus, M. guilio, M. malbaricus*) were collected in the Narmada River. One specimen procured from the Banjar tributary could only be identified to the genus level based on a higher K2P divergence value. To clarify this a few GenBank records for mystus species (*M. malbaricus,* HQ219109.1-HQ219111.1; *M. vitatus,* JN228952.1, JN228053.1) were included in our anaysis. This result shows 11% (SE = 0.03) genetic divergence with an average value of 0.12% (SE = 0.001) between both *M. malabaricus* and *M. vittatus* (Figure 6). The ABGD based analysis partitioned, without ambiguity, these five described species (*M. bleekeri, M. cavasius, M. vitatus, M. guilio, M. malbaricus*) and one suspected putative new/sibling species absolutely without any indentication of being a potential false positive or false negative. This suggests that voucher specimen NF511 represents a putative new species. However, this example, along with most of the suggestions for new species made here are represented by one individual only. Further investigations by the analysis of additional specimens will be necessary in future studies to confirm our observations.

Specimens from seven species considered to be endangered, namely *Labeo dussumieri, Tor tor, Sperata aor, Crossocheilus latius, Heteropneustes fossilis, Puntius sarana and Rhinomugil corsula* [4], were recorded within collections of this study. Of these, *Puntius sarana* showed the highest intraspecific divergence (mean 0.46%, SE = 0.01) as compared to *Heteropneustes fossilis* (0.35%, SE = 0.01), *Sperata aor* (0.34%, SE = 0.03) and *Tor tor* (0.33%, SE = 0.02). The lowest intraspecific divergence (0%) was noted for *Rhinomugil corsula*.

### Genetic diversity and divergence inferred from different sampling sites

**Figure 7:**
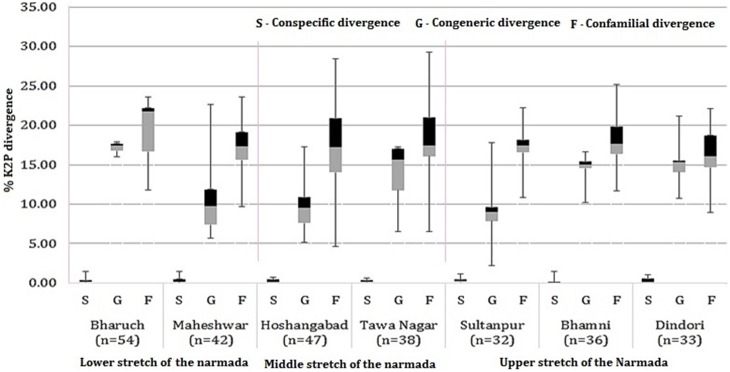
Boxplot showing distribution of conspecific (S) congeneric (G) and confamilial (F) K2P divergence (%) along the selected sampling stations from the River basin analyzed as upper reach, middle and lower reach (segment).

The genetic divergence values for conspecific, congeneric and confamilial from different sampling stations were analyzed by grouping them as stations from the upper stretch, middle stretch and lower stretch regions of the river (Figure 7). The conspecific divergence values between the sampling stations show a uniform distribution (≥0.01; SE = 0.01) while the sampling stations on tributaries had wide range of conspecific divergence values ranging from 0 to 0.16% (SE = 0.01). The divergence values within genera and family were higher at the Bharuch sampling station located at the mouth of river in the Gulf of Cambay (Lower stretch of the river). Congeneric divergence values within the rest of the sampling stations ranged from 8.98% (SE = 0.03) to 15.32% (SE = 0.04), while confamilial values ranged from 16.05% (SE = 0.02) to 17.31% (SE = 0.02). Overall, the upper and lower stretch of the river represent a wide range of divergence values at the genus and family level among the sampling stations located in this part of the Narmada compared to the lower stretch. The decline in the range of genetic divergences on the lower part of the river may be due to fragmentation of the habitat and effects of limiting the fish migration due to large dams such as the Sardar Sarovar and Indira Sagar (Figure 1).

## Discussion

Our study represents the first molecular survey of diversity using COI barcode data of freshwater fishes for whole Narmada River system in India. This includes generation of COI barcodes for 83 fishes out of 85 samples (∼98%) and the inference of five putative new species based on genetic data. These new species represent almost 6% of the known fish diversity of the Narmada. Using these sequences, we showed that the average conspecific K2P genetic divergence was 0.36% (SE = 0.008). This increased to 12.29% (SE = 0.06) at the congeneric level, 17.87% (SE = 0.02) at the confamilial levels and finally to 22.43% (SE = 0.02) within the order (Table 2 and Figure 3). The increase in the levels of genetic divergence observed here with increasing taxonomic levels and can be compared with the ranges obtained in other studies of Indian freshwater fish (1.6%, 7.16%, 16.66% and 25.32% respectively) as described [27], and for Carangid fishes from the Kakinada coast (0.78%, 17.2%, 24.18% and 25.97% respectively) by [28], Indian marine fishes (0.30%, 6.06%, 9.91% and 16% respectively) by [29], and Canadian freshwater fishes (0.27%, 8.37%,15.38% and 20.06% respectively) by [30]. We also observed a ∼40-fold greater level of divergence among congeneric species relative to that of conspecific individuals as compared to an 18 fold increase observed in other studies of Indian freshwater fish [27]. In addition, in our study we observed that the divergence pattern (K2P) varies along the river basin scale (Figure 7), suggesting that for a particular taxonomic group, studies of a complete habitat may bring clearer insights for interpreting such inter and intraspecific divergence values.

In these and other previous studies, considerable effort has been applied to the use of DNA barcoding alone for delineating species boundaries [12], [13], [31]–[33]. Attempts have also been made to establish a standard limit between intra- and inter-species divergence (e.g. 3% of divergence [34] or the 10x rule [35]). However, these could not be generalized to many groups of organisms [36]–[39], [33]. Furthermore, as shown in these studies, intra- and interspecific distances frequently overlap, and visually defining a threshold becomes difficult [16]–[17], [40]–[41]. Also it has been recognized for some time that for cases where there is variation in the mutation rate among genes and species, the use of sequence divergence estimation alone for phylogenetic reconstructions [42]–[44] has considerable limitations, and this may further hinder the use of the DNA barcoding for cataloguing species diversity [45]–[47]. Furthermore, because the data obtained from the COI gene is known to possibly be affected by several biases, ideally it should be combined with the analysis not only of other independent genes, but also with other information such as morphological, geographical or ecological data to clearly delimit species in an integrative framework [48]–[58]. Here we show how barcoding results, combined with the use of the ABGD tool, may act as an independent method for delineating species and boundaries.

In our study 83 species were correctly identified without any overlap for intra and interspecific distances. The data we obtained also showed clear clusters in the NJ tree with sufficient bootstrap support to represent true species. In the case of *Labeo dyochelius* and *Puntius sarana,* the conspecific divergence values were 2.98% and 2.19 respectively, but here the average values are much lower than the 10x threshold of average K2P divergence for congeneric species that has been suggested by Hebert et al. to be able to delimit species using barcode data [35]. Furthermore, a comprehensive review of “barcoded” fishes [59] noted that about 17% of the genetic divergence values among congeneric species were less than the 3% value. They also suggest that if the unknown specimen is more than 2% divergent from a known species, it is very likely that this is a different species (probability greater than 95%). Additionally, hidden diversity and overlooked species have often been detected in various situations [29]–[30], [59]. In summary, it is clear that the threshold limit proposed by Hebert et al. [35] as an indicator of cryptic speciation should be carefully considered for each group. For example, from our data five taxa (*Labeo doychelius, Puntius sarana, Liza spp., Nematolosa spp* and *Mystus spp.)* showed slightly higher divergence values (>2%), but using the ABGD online tool [21] based on a threshold of P = 0.0237 (SE = 0.02), the previously unclear species relationships are nicely partitioned here. This further implies that these previously described “cryptic species” may be evolving independently and radiating from an ancestral population in this river. As in other cases, however, the number of representative sequences in the dataset here was small and may need to be reassessed after collection of additional data.

Overall, when we used our barcode data and the ABGD algorithm with an optimal threshold value to infer any barcode “gap” and to partition the data set to discriminate between all 83 species, groups were formed that corresponded well with those based on morphological species identification (Figure 5). In the use of this algorithm, lowering of the threshold value may cause the splitting of known species into multiple groups, and conversely, increasing this value can completely eliminate any barcode gap. For the taxa from the Narmada River, threshold values lower than P = 0.0237 may create false positives while values greater than P = 0.0237 appear to be false negatives (Figure 5). From this it is clear that the use of barcode based analyses, combined with the use of the ABGD tool, can be used to accurately identify and demarcate species boundaries and to assign unknown individuals to known species.

In our study we initially classified up to 90 species of the Narmada fishes based on morphological characters. These were cross verified by DNA barcoding and analysis using the ABGD tool, and this reduced the species number to 83. We were not able to generate barcodes for two morphological described species (*Parachaeturichthys ocellatus* and *Terapon jarbua*), despite numerous attempts using multiple samples. Of the species we recorded, a number had already reported (43 in reference [6] and 30 in reference [26]). Therefore, 43 species we recorded had previously not been documented in this river. These species, along with those reported in [26] and [6], account for 16% of total diversity of freshwater fishes in India [60], [61].

The limited numbers reported in some of these studies may be due to taxonomic ambiguities based on morphological identifications. Also, out of the three species (*Tor tor, T. putitora, T. khudree*) listed as endagered by [26], we could identify only one, *Tor tor*, in our study. This suggests the possibility of one or more of them being extinct or close to exintiction, although inadequate sampling cannot be ruled out. However, two species listed as threatened (*Notopterus notopterus, Labeo fimbratus*) in the same study were found in abundance in our study (Table S4 and Figure2).

Our study confirms that employing COI barcoding can help in the identification of the majority of fish species in diverse river systems. Increasing use of DNA barcoding can overcome the limitations of morphology based identifications and help identify previously unidentified species by documenting the diversity of COI sequences within currently recognized species. In these cases the identification of taxa may be aided by the partitioning possible through the use of the ABGD online tool to decide on threshold values for identification of putative new species. This use of molecular data should be complementary to morphological analsysis in such endeavours, and the establishment of reliable global COI barcode databases for fishes will help to be able to accurately identify any fish at any stage of the life cycle (such as eggs or larva) or even from small pieces of tissue. This will be a valuable tool in the hands of fisheries managers, ecologists and fish conservators.

## Supporting Information

Table S1 **Gears used for sampling the specimens from Narmada River basin.**
(DOCX)

Table S2 **List of specimens collected from the Narmada River basin with BOLD identification and NCBI Genebank accession numbers.**
(DOCX)

Table S3 **Fish collection details from Narmada River basin.**
(XLSX)

Table S4 **Comparison of fish species from Narmada River basin with earlier studies.**
(XLS)

Methods S1 (DOCX)
