# Supplementary material for: DNA Barcodes for the FIshes of the Narmada, One of India’s Longest Rivers
Source: PLoS One. 2014 Jul 3;9(7):e101460. doi: 10.1371/journal.pone.0101460 (PMC4081587; doi:10.1371/journal.pone.0101460)
Supplement: Table S1 — Gears used for sampling the specimens from Narmada River basin. (DOCX) [file pone.0101460.s001.docx]

Table S1. Nets and gears used for sampling the specimens from Narmada river basin.

| **Nets** | **Samples collected** |
| --- | --- |
| Cast Net | 162 |
| Drag Net | 31 |
| Gill Net | 614 |
| Hook | 11 |
| Shot gun | 2 |
|  | 820 |
