# Supplementary material for: DNA Barcodes for the FIshes of the Narmada, One of India’s Longest Rivers
Source: PLoS One. 2014 Jul 3;9(7):e101460. doi: 10.1371/journal.pone.0101460 (PMC4081587; doi:10.1371/journal.pone.0101460)
Supplement: Table S2 — List of specimens collected from the Narmada River basin with BOLD identification and NCBI Genebank accession numbers. (DOCX) [file pone.0101460.s002.docx]

Table S2. List of specimens collected from Narmada river basin with Identification and Acession numbers

| ***Identification*** | **Sample ID** | **BOLD ID** | **GenBank Acession** |
| --- | --- | --- | --- |
| *Acanthocobitis botia* | NF151 | DBFN305-12 | JX983397 |
| *Acanthocobitis botia* | NF43 | DBFN278-12 | JX983396 |
| *Acanthocobitis botia* | NF425 | DBFN129-11 | JX983398 |
| *Acanthocobitis botia* | NF426 | DBFN130-11 | JX983395 |
| *Acanthopagrus latus* | NFGJ3 | DBFN378-12 | JX983210 |
| *Amblypharyngodon mola* | NF770 | DBFN339-12 | JX983212 |
| *Amblypharyngodon mola* | NF771 | DBFN340-12 | JX983211 |
| *Anabas testudineus* | NF461 | DBFN146-11 | JX983214 |
| *Anabas testudineus* | NF462 | DBFN147-11 | JX983213 |
| *Arius arius* | NF776 | DBFN345-12 | JX983224 |
| *Arius arius* | NF552 | DBFN286-12 | JX983226 |
| *Arius arius* | NF551 | DBFN285-12 | JX983225 |
| *Awaous ocellaris* | NF784 | DBFN352-12 | JX983227 |
| *Awaous ocellaris* | NF785 | DBFN353-12 | JX983228 |
| *Barilius bendelisis* | NF689 | DBFN239-11 | JX983232 |
| *Barilius bendelisis* | NF782 | DBFN350-12 | JX983234 |
| *Barilius bendelisis* | NF783 | DBFN351-12 | JX983229 |
| *Barilius bendelisis* | NF633 | DBFN192-11 | JX983230 |
| *Barilius bendelisis* | NF463 | DBFN148-11 | JX983236 |
| *Barilius bendelisis* | NF634 | DBFN193-11 | JX983231 |
| *Barilius bendelisis* | NF688 | DBFN238-11 | JX983233 |
| *Barilius bendelisis* | NF781 | DBFN349-12 | JX983235 |
| *Boleophthalmus dissumieri* | NF778 | DBFN346-12 | JX983441 |
| *Boleophthalmus dissumieri* | NF779 | DBFN347-12 | JX983442 |
| *Catla catla* | NF661 | DBFN215-11 | JX983238 |
| *Catla catla* | NF696 | DBFN245-11 | JX983237 |
| *Chanda nama* | NF483 | DBFN163-11 | JX983239 |
| *Chanda nama* | NF339 | DBFN106-11 | JX983240 |
| ***Identification*** | **Sample ID** | **BOLD ID** | **GenBank Acession** |
| *Chanda nama* | NF222 | DBFN053-11 | JX983241 |
| *Chanda nama* | NF514 | DBFN185-11 | JX983242 |
| *Channa marulius* | NF22 | DBFN004-11 | JX983243 |
| *Channa marulius* | NF420 | DBFN124-11 | JX983244 |
| *Channa orientalis* | NF503 | DBFN177-11 | JX983245 |
| *Channa orientalis* | NF756 | DBFN370-12 | JX983248 |
| *Channa orientalis* | NF424 | DBFN128-11 | JX983249 |
| *Channa orientalis* | NF504 | DBFN178-11 | JX983246 |
| *Channa orientalis* | NF137 | IOFBI045-11 | JQ667514 |
| *Channa orientalis* | NF765 | DBFN334-12 | JX983247 |
| *Channa punctata* | NF658 | DBFN212-11 | JX983251 |
| *Channa punctata* | NF23 | DBFN005-11 | JX983250 |
| *Cirrhinus mrigala* | NF653 | DBFN208-11 | JX983255 |
| *Cirrhinus mrigala* | NF36 | DBFN010-11 | JX983258 |
| *Cirrhinus mrigala* | NF447 | DBFN141-11 | JX983257 |
| *Cirrhinus mrigala* | NF650 | DBFN205-11 | JX983256 |
| *Cirrhinus mrigala* | NF691 | DBFN241-11 | JX983253 |
| *Cirrhinus mrigala* | NF690 | DBFN240-11 | JX983254 |
| *Cirrhinus mrigala* | NF197 | DBFN044-11 | JX983252 |
| *Cirrhinus reba* | NF104 | DBFN018-11 | JX983268 |
| *Cirrhinus reba* | NF476 | DBFN157-11 | JX983266 |
| *Cirrhinus reba* | NF242 | DBFN071-11 | JX983262 |
| *Cirrhinus reba* | NF345 | DBFN111-11 | JX983270 |
| *Cirrhinus reba* | NF352 | DBFN115-11 | JX983271 |
| *Cirrhinus reba* | NF250 | DBFN077-11 | JX983263 |
| *Cirrhinus reba* | NF254 | DBFN080-11 | JX983264 |
| *Cirrhinus reba* | NF702 | DBFN251-11 | JX983269 |
| *Cirrhinus reba* | NF119 | DBFN031-11 | JX983261 |
| *Cirrhinus reba* | NF108 | DBFN021-11 | JX983265 |
| *Cirrhinus reba* | NF654 | DBFN209-11 | JX983260 |
| *Cirrhinus reba* | NF635 | DBFN194-11 | JX983259 |
| ***Identification*** | **Sample ID** | **BOLD ID** | **GenBank Acession** |
| *Cirrhinus reba* | NF100.1 | DBFN015-11 | JX983292 |
| *Cirrhinus reba* | NF475 | DBFN156-11 | JX983267 |
| *Clarias batrachus* | MPM8 | DBFN390-12 | KF214293 |
| *Clarias batrachus* | MPM6 | DBFN389-12 | KF214294 |
| *Clarias batrachus* | MPM5 | DBFN388-12 | KF214295 |
| *Clarias batrachus* | MPM10 | DBFN387-12 | KF214296 |
| *Clupisoma garua* | NF329 | DBFN096-11 | JX983276 |
| *Clupisoma garua* | NF668 | DBFN220-11 | JX983277 |
| *Clupisoma garua* | NF494 | DBFN170-11 | JX983273 |
| *Clupisoma garua* | NF669 | DBFN221-11 | JX983278 |
| *Clupisoma garua* | NF749 | DBFN368-12 | JX983275 |
| *Clupisoma garua* | NF328 | DBFN095-11 | JX983272 |
| *Clupisoma garua* | NF493 | DBFN169-11 | JX983274 |
| *Crossocheilus latius* | NF397 | DBFN380-12 | KF214297 |
| *Cynoglossus cynoglossus* | NF768 | DBFN337-12 | JX983280 |
| *Cynoglossus cynoglossus* | NF555 | DBFN288-12 | JX983281 |
| *Cynoglossus cynoglossus* | NF554 | DBFN287-12 | JX983282 |
| *Cyprinus carpio* | NF448 | DBFN142-11 | JX983284 |
| *Cyprinus carpio* | NF695 | DBFN244-11 | JX983283 |
| *Devario malabaricus* | NF675 | DBFN227-11 | JX983285 |
| *Devario malabaricus* | NF641 | DBFN199-11 | JX983286 |
| *Devario malabaricus* | NF178 | IOFBI134-11 | JQ667528 |
| *Devario malabaricus* | NF307 | DBFN382-12 | KF214298 |
| *Eleutheronema tetradactylum* | NF576 | DBFN300-12 | JX983438 |
| *Eleutheronema tetradactylum* | NF558 | DBFN291-12 | JX983440 |
| *Eleutheronema tetradactylum* | NF557 | DBFN290-12 | JX983439 |
| *Esomus danricus* | NF499 | DBFN174-11 | JX983290 |
| *Eutropiichthys murius* | NF110 | DBFN023-11 | JX983291 |
| *Gagata cenia* | NF37 | DBFN011-11 | JX983293 |
| *Garra mullya* | NF436 | DBFN134-11 | JX983294 |
| *Garra mullya* | NF437 | DBFN135-11 | JX983295 |
| ***Identification*** | **Sample ID** | **BOLD ID** | **GenBank Acession** |
| *Garra mullya* | NF721 | DBFN266-11 | JX983296 |
| *Glossogobius giuris* | NF515 | DBFN186-11 | JX983307 |
| *Glossogobius giuris* | NF705 | DBFN254-11 | JX983308 |
| *Glossogobius giuris* | NF703 | DBFN252-11 | JX983305 |
| *Glossogobius giuris* | NF647 | DBFN203-11 | JX983304 |
| *Glossogobius giuris* | NF646 | DBFN202-11 | JX983306 |
| *Glossogobius giuris* | NF234 | DBFN063-11 | JX983309 |
| *Glyptothorax lonah* | NF333 | DBFN100-11 | JX983301 |
| *Glyptothorax lonah* | NF332 | DBFN099-11 | JX983297 |
| *Glyptothorax lonah* | NF450 | DBFN144-11 | JX983298 |
| *Glyptothorax lonah* | MGH1 | DBFN366-12 | JX983300 |
| *Glyptothorax lonah* | NF451 | DBFN145-11 | JX983299 |
| *Glyptothorax lonah* | NF622 | DBFN381-12 | KF214299 |
| *Glyptothorax lonah* | MGH2 | DBFN367-12 | JX983302 |
| *Harpadon nehereus* | NFGJ553 | DBFN384-12 | KF214300 |
| *Heteropneustes fossilis* | NF713 | DBFN260-11 | JX983312 |
| *Heteropneustes fossilis* | NF637 | DBFN196-11 | JX983311 |
| *Heteropneustes fossilis* | NF714 | DBFN261-11 | JX983313 |
| *Heteropneustes fossilis* | NF20 | DBFN002-11 | JX983310 |
| *Hilsa toli* | NF582 | DBFN302-12 | JX983316 |
| *Hilsa toli* | NF766 | DBFN335-12 | JX983315 |
| *Hilsa toli* | NF581 | DBFN301-12 | JX983317 |
| *Hilsa toli* | NF560 | DBFN293-12 | JX983314 |
| *Hypophthalmichthys nobilis* | NF27 | DBFN277-12 | JX983319 |
| *Hypophthalmichthys nobilis* | NF26 | DBFN276-12 | JX983318 |
| *Hyporhamphus dussumieri* | NFX02 | DBFN326-12 | JX983320 |
| *Hyporhamphus quoyi* | NF556 | DBFN289-12 | JX983484 |
| *Hyporhamphus quoyi* | NF593 | DBFN304-12 | JX983485 |
| *Labeo bata* | NF19 | DBFN001-11 | JX983321 |
| *Labeo bata* | NF731 | DBFN383-12 | KF214301 |
| *Labeo bata* | NF240 | DBFN069-11 | JX983322 |
| ***Identification*** | **Sample ID** | **BOLD ID** | **GenBank Acession** |
| *Labeo boggut* | NF701 | DBFN250-11 | JX983329 |
| *Labeo boggut* | NF99 | DBFN014-11 | JX983324 |
| *Labeo boggut* | NF636 | DBFN195-11 | JX983328 |
| *Labeo boggut* | NF434 | DBFN133-11 | JX983327 |
| *Labeo boggut* | NF433 | DBFN132-11 | JX983323 |
| *Labeo boggut* | NF707 | DBFN255-11 | JX983330 |
| *Labeo boggut* | NF708 | DBFN256-11 | JX983331 |
| *Labeo boggut* | NF349 | DBFN114-11 | JX983326 |
| *Labeo boggut* | NF348 | DBFN113-11 | JX983325 |
| *Labeo calbasu* | NF660 | DBFN214-11 | JX983338 |
| *Labeo calbasu* | NF239 | DBFN068-11 | JX983339 |
| *Labeo calbasu* | NF694 | DBFN243-11 | JX983337 |
| *Labeo calbasu* | NF200 | DBFN047-11 | JX983340 |
| *Labeo dussumieri* | NF252 | DBFN079-11 | JX983347 |
| *Labeo dussumieri* | NF260 | DBFN086-11 | JX983346 |
| *Labeo dussumieri* | NF700 | DBFN249-11 | JX983348 |
| *Labeo dussumieri* | NF235 | DBFN064-11 | JX983349 |
| *Labeo dyocheilus* | NF251 | DBFN078-11 | JX983343 |
| *Labeo dyocheilus* | NF724 | DBFN267-11 | JX983342 |
| *Labeo dyocheilus* | NF676 | DBFN228-11 | JX983341 |
| *Labeo dyocheilus* | NF136 | DBFN034-11 | JX983344 |
| *Labeo fimbriatus* | NF677 | DBFN229-11 | JX983352 |
| *Labeo fimbriatus* | NF353 | DBFN116-11 | JX983351 |
| *Labeo fimbriatus* | NF354 | DBFN117-11 | JX983350 |
| *Labeo dussumieri* | NF236 | DBFN065-11 | JX983345 |
| *Labeo rohita* | NF466 | DBFN151-11 | JX983334 |
| *Labeo rohita* | NF692 | DBFN242-11 | JX983336 |
| *Labeo rohita* | NF659 | DBFN213-11 | JX983332 |
| *Labeo rohita* | NF35 | DBFN009-11 | JX983333 |
| *Labeo rohita* | NF34 | DBFN008-11 | JX983335 |
| *Lates calcarifer* | NF587 | DBFN303-12 | JX983354 |
| ***Identification*** | **Sample ID** | **BOLD ID** | **GenBank Acession** |
| *Liza klunzingeri* | NF762 | DBFN331-12 | JX983356 |
| *Liza klunzingeri* | NF761 | DBFN330-12 | JX983357 |
| *Liza klunzingeri* | NF760 | DBFN329-12 | JX983355 |
| *Liza sp.* | NF550 | DBFN284-12 | JX983495 |
| *Liza sp.* | NF565 | DBFN295-12 | JX983496 |
| *Macrognathus pancalus* | NF423 | DBFN127-11 | JX983358 |
| *Mastacembelus armatus* | NF421 | DBFN125-11 | JX983364 |
| *Mastacembelus armatus* | NF138 | DBFN036-11 | JX983363 |
| *Mastacembelus armatus* | NF139 | DBFN037-11 | JX983359 |
| *Mastacembelus armatus* | NF249 | DBFN076-11 | JX983360 |
| *Mastacembelus armatus* | NF265 | DBFN091-11 | JX983365 |
| *Mastacembelus armatus* | NF336 | DBFN103-11 | JX983361 |
| *Mastacembelus armatus* | NF337 | IOFBI130-11 | JQ667549 |
| *Mastacembelus armatus* | NF422 | DBFN126-11 | JX983362 |
| *Megalops cyprinoides* | NF262 | DBFN088-11 | JX983366 |
| *Mugil cephalus* | NF569 | DBFN297-12 | JX983367 |
| *Mugil cephalus* | NF549 | DBFN283-12 | JX983368 |
| *Mugil cephalus* | NF574 | DBFN299-12 | JX983369 |
| *Mystus bleekeri* | NF682 | DBFN233-11 | JX983373 |
| *Mystus bleekeri* | NF323 | DBFN312-12 | JX983372 |
| *Mystus bleekeri* | NF164 | DBFN307-12 | JX983371 |
| *Mystus bleekeri* | NF764 | DBFN333-12 | JX983370 |
| *Mystus bleekeri* | NF716 | DBFN263-11 | JX983376 |
| *Mystus bleekeri* | NF715 | DBFN262-11 | JX983374 |
| *Mystus bleekeri* | NF750 | DBFN369-12 | JX983375 |
| *Mystus cavasius* | NF685 | DBFN235-11 | JX983382 |
| *Mystus cavasius* | NF334 | DBFN101-11 | JX983380 |
| *Mystus cavasius* | NF399 | DBFN120-11 | JX983383 |
| *Mystus cavasius* | NF325 | DBFN092-11 | JX983377 |
| *Mystus cavasius* | NF492 | DBFN168-11 | JX983379 |
| *Mystus cavasius* | NF400 | DBFN121-11 | JX983381 |
| ***Identification*** | **Sample ID** | **BOLD ID** | **GenBank Acession** |
| *Mystus gulio* | NFGJ557 | DBFN391-12 | JX983439 |
| *Mystus sp.* | NF511 | DBFN183-11 | JX983384 |
| *Mystus vittatus* | NF510 | DBFN182-11 | JX983385 |
| *Nandus nandus* | NF673 | DBFN225-11 | JX983388 |
| *Nandus nandus* | NF219 | DBFN050-11 | JX983391 |
| *Nandus nandus* | NF220 | DBFN051-11 | JX983387 |
| *Nandus nandus* | NF464 | DBFN149-11 | JX983392 |
| *Nandus nandus* | NF32 | DBFN006-11 | JX983386 |
| *Nandus nandus* | NF343 | DBFN109-11 | JX983389 |
| *Nandus nandus* | NF672 | DBFN224-11 | JX983390 |
| *Nemacheilus montana* | DNN2 | DBFN364-12 | JX983394 |
| *Nemacheilus montana* | DNN1 | DBFN363-12 | JX983393 |
| *Nematalosa* | NF257 | DBFN379-12 | KF214303 |
| *Nematalosa nasus* | NF772 | DBFN341-12 | JX983399 |
| *Nematalosa nasus* | NF559 | DBFN292-12 | JX983401 |
| *Notopterus notopterus* | NF657 | DBFN211-11 | JX983408 |
| *Notopterus notopterus* | NF228 | DBFN059-11 | JX983407 |
| *Notopterus notopterus* | NF326 | DBFN093-11 | JX983402 |
| *Notopterus notopterus* | NF327 | DBFN094-11 | JX983410 |
| *Notopterus notopterus* | NF198 | DBFN045-11 | JX983409 |
| *Notopterus notopterus* | NF678 | DBFN230-11 | JX983404 |
| *Notopterus notopterus* | NF256 | DBFN082-11 | JX983403 |
| *Notopterus notopterus* | NF112 | DBFN024-11 | JX983405 |
| *Notopterus notopterus* | NF229 | DBFN060-11 | JX983406 |
| *Ompok bimaculatus* | NF331 | DBFN098-11 | JX983420 |
| *Ompok bimaculatus* | NF649 | DBFN204-11 | JX983412 |
| *Ompok bimaculatus* | NF501 | DBFN175-11 | JX983411 |
| *Ompok bimaculatus* | NF603 | DBFN316-12 | JX983418 |
| *Ompok bimaculatus* | NF666 | DBFN219-11 | JX983421 |
| *Ompok bimaculatus* | NF602 | DBFN315-12 | JX983419 |
| *Ompok bimaculatus* | NF109 | DBFN022-11 | JX983417 |
| ***Identification*** | **Sample ID** | **BOLD ID** | **GenBank Acession** |
| *Ompok bimaculatus* | NF709 | DBFN257-11 | JX983415 |
| *Ompok bimaculatus* | NF247 | DBFN074-11 | JX983416 |
| *Ompok bimaculatus* | NF330 | DBFN097-11 | JX983414 |
| *Ompok bimaculatus* | NF502 | DBFN176-11 | JX983413 |
| *Oreochromis mossambicus* | NF775 | DBFN344-12 | JX983422 |
| *Osteobrama cotio* | NF631 | DBFN190-11 | JX983432 |
| *Osteobrama cotio* | NF223 | DBFN054-11 | JX983424 |
| *Osteobrama cotio* | NF224 | DBFN055-11 | JX983425 |
| *Osteobrama cotio* | NF341 | DBFN108-11 | JX983428 |
| *Osteobrama cotio* | NF340 | DBFN107-11 | JX983427 |
| *Osteobrama cotio* | NF513 | DBFN184-11 | JX983431 |
| *Osteobrama cotio* | NF484 | DBFN164-11 | JX983429 |
| *Osteobrama cotio* | NF485 | DBFN165-11 | JX983430 |
| *Osteobrama cotio* | NF632 | DBFN191-11 | JX983423 |
| *Osteobrama cotio* | NF786 | DBFN354-12 | JX983433 |
| *Osteobrama cotio* | NF259 | DBFN085-11 | JX983426 |
| *Panna microdon* | NF573 | DBFN298-12 | JX983436 |
| *Pellona ditchela* | NFGH569 | DBFN385-12 | KF214304 |
| *Protonibea diacanthus* | NF48 | DBFN280-12 | JX983434 |
| *Protonibea diacanthus* | NF50 | DBFN282-12 | JX983435 |
| *Pseudambassis ranga* | NF221 | IOFBI127-11 | JQ667560 |
| *Puntius sarana* | NF471 | DBFN154-11 | JX983449 |
| *Puntius sarana* | NF264 | DBFN090-11 | JX983457 |
| *Puntius sarana* | NF355 | DBFN118-11 | JX983460 |
| *Puntius sarana* | NF356 | DBFN119-11 | JX983458 |
| *Puntius sarana* | NF217 | DBFN048-11 | JX983459 |
| *Puntius sarana* | NF199 | DBFN046-11 | JX983448 |
| *Puntius sarana* | NF140 | DBFN038-11 | JX983452 |
| *Puntius sarana* | NF115 | DBFN027-11 | JX983444 |
| *Puntius sarana* | NF98 | IOFBI049-11 | JQ667570 |
| *Puntius sarana* | NF465 | DBFN150-11 | JX983453 |
| ***Identification*** | **Sample ID** | **BOLD ID** | **GenBank Acession** |
| *Puntius sarana* | NF467 | DBFN152-11 | JX983451 |
| *Puntius sarana* | NF468 | DBFN153-11 | JX983450 |
| *Puntius sarana* | NF201 | DBFN309-12 | JX983446 |
| *Puntius sarana* | NF202 | DBFN310-12 | JX983447 |
| *Puntius sarana* | NF203 | DBFN311-12 | JX983445 |
| *Puntius sarana* | NF651 | DBFN206-11 | JX983454 |
| *Puntius sarana* | NF652 | DBFN207-11 | JX983455 |
| *Puntius sarana* | NF769 | DBFN338-12 | JX983456 |
| *Puntius sophore* | NF490 | DBFN167-11 | JX983465 |
| *Puntius sophore* | NF671 | DBFN223-11 | JX983463 |
| *Puntius sophore* | NF489 | DBFN166-11 | JX983464 |
| *Puntius sophore* | NF472 | DBFN155-11 | JX983462 |
| *Puntius sophore* | NF439 | DBFN137-11 | JX983461 |
| *Puntius ticto* | NF638 | DBFN197-11 | JX983473 |
| *Puntius ticto* | NF218 | DBFN049-11 | JX983468 |
| *Puntius ticto* | NF680 | DBFN232-11 | JX983466 |
| *Puntius ticto* | NF679 | DBFN231-11 | JX983467 |
| *Puntius ticto* | NF261 | DBFN087-11 | JX983469 |
| *Puntius ticto* | NF232 | DBFN062-11 | JX983474 |
| *Puntius ticto* | NF643 | DBFN200-11 | JX983471 |
| *Puntius ticto* | NF644 | DBFN201-11 | JX983472 |
| *Puntius ticto* | NF720 | DBFN265-11 | JX983470 |
| *Rasbora daniconius* | NF445 | DBFN139-11 | JX983475 |
| *Rasbora daniconius* | NF446 | DBFN140-11 | JX983478 |
| *Rasbora daniconius* | NF780 | DBFN348-12 | JX983476 |
| *Rasbora daniconius* | NF757 | DBFN371-12 | JX983477 |
| *Rasbora rasbora* | NF717 | DBFN264-11 | JX983479 |
| *Rasbora rasbora* | NF227 | DBFN058-11 | JX983481 |
| *Rasbora rasbora* | NF498 | DBFN173-11 | JX983480 |
| *Rhinomugil corsula* | NF568 | DBFN296-12 | JX983483 |
| *Rhinomugil corsula* | NF759 | DBFN328-12 | JX983482 |
| ***Identification*** | **Sample ID** | **BOLD ID** | **GenBank Acession** |
| *Rhinomugil corsula* | NFGJ573 | DBFN393-12 | KF214305 |
| *Salmophasia boopis* | NF225 | DBFN056-11 | JX983492 |
| *Salmostoma bacalia* | NF495 | DBFN171-11 | JX983486 |
| *Salmostoma bacalia* | NF670 | DBFN222-11 | JX983488 |
| *Salmostoma bacalia* | NF191 | DBFN039-11 | JX983491 |
| *Salmostoma bacalia* | NF344 | DBFN110-11 | JX983487 |
| *Salmostoma bacalia* | NF497 | DBFN172-11 | JX983489 |
| *Salmostoma bacalia* | NF192 | DBFN040-11 | JX983490 |
| *Scatophagus argus* | NF578 | DBFN325-12 | JX983493 |
| *Scomberoides lysan* | NF46 | DBFN279-12 | JX983494 |
| *Sperata aor* | NF664 | DBFN218-11 | JX983217 |
| *Sperata aor* | NF195 | DBFN042-11 | JX983215 |
| *Sperata aor* | NF335 | DBFN102-11 | JX983218 |
| *Sperata aor* | NF103 | DBFN017-11 | JX983216 |
| *Sperata seenghala* | NF449 | DBFN143-11 | JX983219 |
| *Sperata seenghala* | NF663 | DBFN217-11 | JX983220 |
| *Sperata seenghala* | NF196 | DBFN043-11 | JX983222 |
| *Sperata seenghala* | NF255 | DBFN081-11 | JX983221 |
| *Strongylura strongylura* | NF49 | DBFN281-12 | JX983497 |
| *Strongylura strongylura* | NFGJ555 | DBFN392-12 | KF214306 |
| *Thryssa malabarica* | NF52 | DBFN327-12 | JX983289 |
| *Thryssa malabarica* | NF564 | DBFN294-12 | JX983287 |
| *Thryssa malabarica* | NF763 | DBFN332-12 | JX983288 |
| *Tor tor* | NF735 | DBFN317-12 | JX983504 |
| *Tor tor* | NF169 | DBFN308-12 | JX983499 |
| *Tor tor* | MT1 | DBFN362-12 | JX983503 |
| *Tor tor* | NF699 | DBFN248-11 | JX983502 |
| *Tor tor* | NF662 | DBFN216-11 | JX983500 |
| *Tor tor* | DNP1 | DBFN365-12 | JX983501 |
| *Tor tor* | RPT | DBFN356-12 | JX983505 |
| *Upeneus vittatus* | NFGJ2 | DBFN377-12 | JX983506 |
| ***Identification*** | **Sample ID** | **BOLD ID** | **GenBank Acession** |
| *Wallago attu* | NF710 | DBFN258-11 | JX983507 |
| *Wallago attu* | NF683 | DBFN234-11 | JX983508 |
| *Wallago attu* | BW1 | DBFN372-12 | JX983509 |
| *Wallago attu* | NF674 | DBFN226-11 | JX983510 |
| *Xenentodon cancila* | RPX1 | DBFN357-12 | JX983512 |
| *Xenentodon cancila* | JBX1 | DBFN359-12 | JX983511 |
| *Xenentodon cancila* | MMX1 | DBFN358-12 | JX983513 |
| *Glossogobius giuris* | NF704 | DBFN253-11 | JX983303 |
| *Labeo dussumieri* | NF245 | DBFN072-11 | JX983353 |
| *Xenentodon cancila* | HX1.1 | DBFN360-12 | JX983514 |
