# Supplementary material for: DNA Barcodes for the FIshes of the Narmada, One of India’s Longest Rivers
Source: PLoS One. 2014 Jul 3;9(7):e101460. doi: 10.1371/journal.pone.0101460 (PMC4081587; doi:10.1371/journal.pone.0101460)
Supplement: Methods S1 — (DOCX) [file pone.0101460.s005.docx]

**Methods S1**

*DNA isolation*

A fin clip was removed from each fish for DNA analysis and the remainder of the specimen was preserved in absolute ethanol. Morphological identifications were based on information in FishBase (www.fishbase.org) and standard identification references [1-2]. We collected 820 specimens, and on average two to three individuals per species were selected for sequence analysis. Multiple specimens of some species from the same sampling location were excluded from further processing, except those showing morphological variation. This included 18 specimens of *Puntius sarana*, a species with very high phenotypic plasticity.

Genomic DNA was extracted from portions of the finclips using the Wizard Genomic DNA isolation kit (Promega). The resultant DNA extracts were diluted to 20ng/µl and stored at -20^O^C for future use.

*PCR and Sequencing*

The barcode region of the COI gene was amplified after testing several primer sets and PCR conditions. The primers showing highest amplification efficiency were FF2d-FR1d and C_FishF1t1-C_FishR1t1 fish cocktail primer pair [3] tailed with M13 to facilitate sequencing [4-5]. PCR reactions were performed using Kapa biosystems kit in 96-well plates. The reaction master mix consisted of 9.6 µl of 10% trehalose, 7.0 µl H_2_O, 2.5 µl 10X PCR buffer ‘B’, 0.8 µl 50mM MgCl_2_, 2.0 µl 2.5mM DNTP, 1.0 µl 10mM forward and reverse primers each and 0.1 µl taq polymerase (5 units/ µl). PCR included an initial step of 2 min at 95⁰C and 35 cycles of 30 sec at 94⁰C, 40 sec at 52⁰C, and 1 min at 72⁰C, with a final extension at 72⁰C for 10 min. Amplicons were visualized on 1.2% agarose gels before unincorporated nucleotides and residual primers were removed with Exo-SAP. M13 forward and reverse primers were used to generate a bidirectional sequence record for each amplicon. A1/16 dilution of BigDye® Terminator v.3.1 Cycle Sequencing Kit (Applied Biosystems, Inc.) was used for sequencing. The cycle sequencing products were cleaned by ethanol precipitation and the template was dissolved in HiDi formamide before analysis on an ABI 3130 Genetic analyzer (Applied Biosystems, Inc.).

**References**

1. Day F (1889) The fauna of British India, including Ceylon and Burma. Published under the authority of the secretary of state for India in council. Edited by W. T. Blaxford. Fishes. Vol. I & II.
2. Talwar PK, Jhingran AG (1991) Inland fishes of India and Adjacent Countries. New Delhi: Oxford & IBH Pub. Co. 1158 p.
3. Jayaram KC (1981) The fresh water fishes of India, Pakistan, Bangladesh, Burma and Sri Lanka– A Handbook. Zoological Survey of India, Calcutta. 475 p.
4. Ivanova NV, Zemlak TS, Hanner R, Hebert PDN (2007) Universal primer cocktails for fish DNA barcoding. Mol. Eco. Notes*.* 7(4): 554-558.
5. Messing J (1983) New M13 vectors for cloning. Methods Enzymol. 101:20–78.
